# Anthelmintic and relaxant activities of *Verbascum Thapsus *Mullein

**DOI:** 10.1186/1472-6882-12-29

**Published:** 2012-03-30

**Authors:** Niaz Ali, Syed Wadood Ali Shah, Ismail Shah, Ghayour Ahmed, Mehreen Ghias, Imran Khan, Waqar Ali

**Affiliations:** 1 Department of Pharmacology, Institute of Basic Medical Sciences, Khyber Medical University, Peshawar, KPK, Pakistan; 2 Department of Pharmacy, University of Malakand, Chakdara, Dir, KPK, Pakistan; 3 Department of Biotechnology, University of Malakand, Chakdara, Dir, KPK, Pakistan; 4 Pharm-D Scholar, Department of Pharmacy, Abasyn University, Peshawar, KPK, Pakistan

## Abstract

**Background:**

*Verbascum thapsus *is used in tribal medicine as an antispasmodic, anti-tubercular agent and wormicide. In this study, we investigated the antispasmodic and anthelmintic activities of crude aqueous methanolic extract of the plant.

**Methods:**

*V. thapsus *extracts were tested against roundworms (*Ascaridia galli*) and tapeworms (*Raillietina spiralis*). Each species of worm was placed into a negative control group, an albendazole treatment group, or a *V. thapsus *treatment group, and the time taken for paralysis and death was determined. In addition, relaxation activity tests were performed on sections of rabbit's jejunum. Plant extracts were tested on KCl-induced contractions and the relaxation activities were quantified against atropine. *V. thapsus *calcium chloride curves were constructed to investigate the mode of action of the plant extracts.

**Results:**

We detected flavonoids, saponins, tannins, terpenoids, glycosides, carbohydrates, proteins, fats and fixed oils in *V. thapsus*. For both species of worm, paralysis occurred fastest at the highest concentration of extract. The relative index values for paralysis in *A. galli *were 4.58, 3.41 and 2.08, at concentrations of 10, 20 and 40 mg/ml of plant extract, respectively. The relative index for death in *A. galli *suggested that *V. thapsus *extract is wormicidal at high concentration. Similarly, the relative indexes for paralysis and death in *R. spiralis *suggested that the extract is a more potent wormicidal agent than albendazole. The mean EC_50 _relaxation activity values for spontaneous and KCl induced contractions were 7.5 ± 1.4 mg/ml (6.57-8.01, n = 6) and 7.9 ± 0.41 mg/ml (7.44-8.46, n = 6), respectively. The relaxation activity of the extract was 11.42 ± 2, 17.0 ± 3, 28.5 ± 4, and 128.0 ± 7% of the maximum observed for atropine at corresponding concentrations. The calcium chloride curves showed that *V. thapsus *extracts (3 mg/ml), had a mean EC_50 _(log molar [calcium]) value of -1.9 ± 0.06 (-1.87 - -1.98, n = 6) *vs*. control EC_50 _= -2.5 ± 0.12 (-2.37 - -2.56, n = 6), whereas the verapamil (0.1 μM) EC_50 _was -1.7 ± 0.1 (-1.6 - -1.8, n = 6) *vs*. control EC_50 _= -2.4 ± 0.09 (-2.3 - -2.47, n = 5).

**Conclusions:**

Our results suggest that *V. thapsus*, which is currently used by some tribes in the Malakand region of Pakistan, has anthelmintic and antispasmodic value.

## Background

The Scrophulariaceae are members of the Figwort family of herbs and shrubs. They comprise 269 genera and 5100 species, all of which are located in temperate and tropical mountainous areas [1]. The genus *Verbascum*, which is also a member of the Figwort family, is represented by 360 global species [2]. Plants within this genus are widely used in folklore medicine [3], and have, therefore, potential pharmacological importance. *Verbascum *leaves and flowers are reported to have expectorant, mucolytic and demulcent properties and are used in traditional Turkish medicine to treat respiratory conditions such as bronchitis, dry coughs, tuberculosis and asthma. Plants within this genus are also used to treat hemorrhoids, rheumatic pain, superficial fungal infections, wounds and diarrhea. Such plants have inhibitory activities against murine lymphocytic leukemia and influenza viruses A2 and B [4]. In addition, *Verbascum phlomoides *contains an iridoid ester glycoside (known as specioside), the caffeic acid esters verbascoside and forsythoside B, and the saponins desrhamnosyl verbascosaponin [5] and verbascoside [3]. *V. phlomoides *and *V. densiflorum *also contain the iridoid compounds aucubin, catalpol, 6-*O*-β-D-xylopyranosylaucubin and saccatoside. *V. densiflorum *contains the iridoids aucubin and catalpol, as well as harpagide, harpagide acetate, and 6-*O*-(4″-*p*-methoxy-*trans*-cinnamoyl)-α-L-rhamnopyranosyl catalpol [6]. Extracts of *V. gypsicola *and *V. sinuatum *have demonstrated antimicrobial activities [4,7].

*Verbascum *species have numerous medicinal properties. For example, the leaves, flowers and roots have been used for treating fevers and bleeding from the lungs. The same parts have also been used as an astringent [8]. Whilst the whole plant has been used to treat diarrhea and dysentery, and also as an analgesic and antiseptic, the paste obtained from leaves and flowers is used for coughs and pulmonary diseases; the seeds have narcotic properties [9-11]. Powered leaves in the form of a poultice are used to relieve joints pain and to soften boils [12]. Dried leaves of *V. thapsus*, however, are smoked for mental relaxation, whilst tea made from its leaves is used to treat colds and dysentery. It is believed that smoke from the plant can drive away ghosts from children [13]. The product produced from decoction of its dried leaves and flowers is used to treat sore throats, bronchitis and abdominal pain, and can act as an expectorant and sedative [14]. The leaves and flowers have antispasmodic properties and are used as an expectorant for bronchitis, tuberculosis and other respiratory ailments. *V. thapsus *leaves are also smoked to ease chest complaints and asthma [15].

The plant is used by some tribes within the Malakand region of Pakistan; hence we were interested in investigating its potential medicinal properties further. This research, therefore, was conducted to investigate: 1) the antispasmodic activity of *V. thapsus *in the context of abdominal pain, and 2) the anthelmintic activity of *V. thapsus *.

## Methods

### Collection, identification and preparation of plant material

Fresh aerial growing parts of *V. thapsus *(300 g) were collected from the hills near to the Chakdara campus of the University of Malakand, Pakistan. Professor Jehandar Shah, Vice Chancellor of the Shaheed Benazir Bhutto University in Pakistan located and identified the plants. A voucher specimen designated Vt-01-2009 has been submitted to the herbarium of the University of Malakand. Fresh aerial sections of the plants were gently washed with distilled water, dried in the shade, crushed and macerated in 1.0 L commercial grade methanol for 3-4 days, then filtered. This process was repeated three times prior to combining the filtrates and evaporating them under reduced pressure using a rotary evaporator at 40°C until a solvent free semisolid extract was obtained (yield = 9.9%).

### Animals and drugs

Anthelmintic activity was determined using adult roundworms (*Ascaridia galli*) and tapeworms (*Raillietina spiralis*), which were identified by a veterinary practitioner and zoologist from the Biotechnology Department of the University of Malakand. Fresh worm-infested intestines obtained from fowls (chickens) were collected from a nearby slaughter house in Chakdara. The tissues were kept in normal saline. Tapeworms and roundworms were isolated from the intestines. Parasites were maintained in normal saline during the experimental period. The average length of the earthworms was 6-8 cm; tapeworms were 6-7.8 cm in length, whilst roundworms were 4.8-7 cm long. Albendazole (Glaxo Smith Kline) was used as the standard anthelmintic drug [16].

Unless specified, analytical grade chemicals (E. Merck, Germany) were used throughout these experiments. Acetylcholine was purchased from BDH Chemicals, Poole, England. All solutions were freshly prepared in distilled water on experimental days. Rabbits were purchased from a local market and bred at the University of Malakand animal house. Rabbits were treated according to the principles of the "Animals Byelaws 2008 of the University of Malakand (Scientific Procedures Issue-I)". The Ethical Committee of the Department of Pharmacy, constituted under the approved Animals Byelaws 2008 of the University of Malakand, endorsed the study protocols.

### Preliminary phytochemical screening

Preliminary phytochemical tests for the powdered materials and extracts of *V. thapsus *were conducted to determine the presence of flavonoids, saponins, tannins, glycosides, cardiac glycosides, carbohydrates, proteins, and sterols according to standard procedures [17-19].

### Anthelmintic activity

The anthelmintic activity of *V. thapsus *was determined using the method described by Ajaiyeoba, et al., with fresh adult roundworms and tapeworms [20-23]. Test samples of the aqueous methanolic extract of *V. thapsus *(Vt.Cr) were prepared at concentrations of 10, 20 and 40 mg/ml in normal saline. Six worms of approximately equal size of both species of were placed in a petri dish containing 25 ml of the test solutions of *V. thapsus*. Solutions of albendazole (10 mg/ml), or distilled water, each containing six test worms were used as the standard and negative control, respectively. All test solutions and standards were freshly prepared at the time of the experiments. The time taken for paralysis in the worms to develop was recorded. Paralysis was defined as the time when all movement had stopped, except for when the worms were shaken vigorously. The time of death was defined as when no movement occurred upon vigorous shaking or dipping the worms into warm water (50°C). All experiments were conducted in quadruplicate.

### Recording electrophysiological and electro pharmacological effects

A force transducer (MLT 0210/A) connected to Power Lab ADInstruments (Australia) was used to record tissue responses. The settings used were 5 Hz × 10 gain (input 1) @40/S, low pass, range 20 mv.

### Effects on rabbit jejunum

Rabbits of either sex (average weight = 1.8 ± 0.2 kg) were purchased from a local market. They were bred in the animal house at the University of Malakand. Rabbits were starved for 24 hours prior to starting the experiments, but had free access to water. Their abdomens were surgically opened after cervical dislocation. Sections of jejunum were removed and maintained in petri dishes constantly aerated with carbogen (95% oxygen: 5% Carbon dioxide) gas [24,25]. Rabbits' jejunum preparations (1-1.5 cm) were mounted in 10 ml tissue baths containing Tyrode's solution (2.68 mM KCl, 136.9 mM NaCl, 1.05 mM MgCl_2_, 11.90 mM NaHCO_3_, 0.42 mM NaH_2 _PO_4_, 1.8 mM CaCl_2 _and 5.55 mM glucose). Tissues were maintained at 37 ± 1 degree Celsius, with constant bubbling of carbogen gas. The tissues were stabilized for 20 minutes. After stabilization resulting in reproducible tissue responses, the Vt.Cr was tested at concentrations 0.01, 0.03, 0.1, 0.3, 1.0, 3.0, 5.0 and 10.0 mg/ml [24,25]. The degree to which Vt.Cr induced relaxant responses in the tissue samples was quantified against 1.0 μM atropine.

### Effects on KCl induced contractions

While attempting to explain the possible mode of action of *V. thapsus*, concentrations of its aqueous methanolic extract (i.e. 0.01, 0.03, 0.1, 0.3. 1.0, 3.0, 5.0 and 10.0 mg/ml) were tested on the sustained contractions induced by KCl (80 mM) on the jejunum preparations [24,25]. All tissue samples had previously been stabilized in normal Tyrode's solution for at least 30 minutes.

### Effects of *V. thapsus *on calcium chloride curves

To confirm the possible mode of action of *V. thapsus *relaxation activity, control calcium chloride curves were constructed using decalcified tissues. Briefly, tissue samples were stabilized in 10 ml tissue baths containing Tyrode's normal solution. The tissues were then exposed to K-normal Tyrode's solution followed by K-rich Tyrode's solution (50 mM KCl, 91.04 mM NaCl, 1.05 mM MgCl_2_, 11.90 mM NaHCO_3_, 0.42 mM NaH_2 _PO_4_, 5.55 mM glucose and 0.1 mM EDTA) [24-26]. This led to decalcification of the tissues. Tissues were constantly bubbled with carbogen gas at 37 ± 1°C. The tissues were calcified using 1 × 10^-4 ^- 256 × 10^-4 ^molar concentrations of calcium. Standard control curves were constructed. Tissues were treated with Vt.Cr at a concentration of 3.0 mg/ml or 5.0 mg/ml. Following a one hour incubation period, calcium chloride curves were constructed. Similarly, control curves for verapamil (0.1 and 0.3 μM) were constructed. The EC_50 _values of the crude methanolic extract of *V. thapsus *were compared against the respective controls to determine if a right shift had occurred in the curves.

### Statistical interpretation of the data

Chart 5 was used to interpret the electrophysiological data (ADInstruments, Australia). Student's t-tests were used to determine statistical significance at 95% confidence intervals. *P *values less than or equal to 0.05 were considered statistically significant. Graph Pad Prism and XL sheet were used to draw the curves. EC_50 _values and the standard error of mean (SEM) were calculated at 95% confidence intervals.

## Results and discussion

Phytochemical testing of *V. thapsus *revealed the presence of flavonoids, saponins, tannins, terpenoids, glycosides, carbohydrates, proteins, fats and fixed oils in the plant extract.

The results of the anthelmintic activity tests are summarized in Table 1. It is clear that at the higher concentrations, Vt.Cr produced paralysis in the test worms. Paralysis occurred faster at higher concentrations of Vt.Cr (i.e. 25 ± 2.3 minutes at 40 mg/ml) in in *A. galli*. A similar trend was observed for *R. spiralis *(28 ± 23 minutes at 40 mg/ml Vt.Cr). The relative index values obtained for *A. galli *were, 4.58, 3.41 and 2.08, at concentrations of 10, 20 and 40 mg/ml Vt.Cr, respectively, *vs*. albendazole (10 mg/ml). In contrast, the relative index for death in *A. galli *was 1.09 (40 mg/ml); this suggests that the anthelmintic activity of Vt.Cr at high concentration is comparable with albendazole. Strikingly, we found that the relative index for paralysis and death in *R. spiralis *were 1.33 and 0.824, respectively (40 mg/ml). This result demonstrates that the *V. thapsus *extract has a more potent wormicidal activity than albendazole against *R. spiralis*. It is hoped that these findings might prove useful for activity guided isolation of bioactive compounds from *V. thapsus*.

**Table 1:** Anthelmintic activity of *Verbascum thapsus *extracts against *Ascaridia galli *and *Raillietina spiralis*

S. No	Groups	Concentrations (mg/ml of normal saline)	Test organisms							
			
			*Ascaridia galli*				*Raillietina spiralis*			
			
			Paralysis		Death		Paralysis		Death	
			
			Time taken (Mean ± SEM, n = 4)	Relative index (P)	Time taken (Mean ± SEM, n = 4)	Relative index (D)	Time taken (Mean ± SEM, n = 4)	Relative index (P)	Time taken (Mean ± SEM, n = 4)	Relative index (D)
	Negative Control	0	----	----	----	----	----	----	----	----

1	*Verbascum thapsus*	10	55 ± 3.4	4.58	81 ± 4	1.97	58 ± 3.7	2.76	86 ± 5	1.50
		
2		20	41 ± 3.6	3.41	63 ± 4.3	1.53	37 ± 2.6	1.76	64 ± 4.8	1.12
		
3		40	25 ± 2.3	2.08	45 ± 4	1.09	28 ± 3	1.33	47 ± 3.7	0.824

4	Albendazole*	10	12 ± 1.8	1	41 ± 3.7	1	21 ± 2.5	1	57 ± 4	1

Key: Relative index (P) denotes the time taken for paralysis to occur using *V. thapsus *extract/the time taken for paralysis to occur using the standard *. Relative index (D) denotes the time taken for death to occur using *V. thapsus *extract/the time taken for death to occur using the standard *). Experimental times were recorded in minutes.

**Figure 1:**
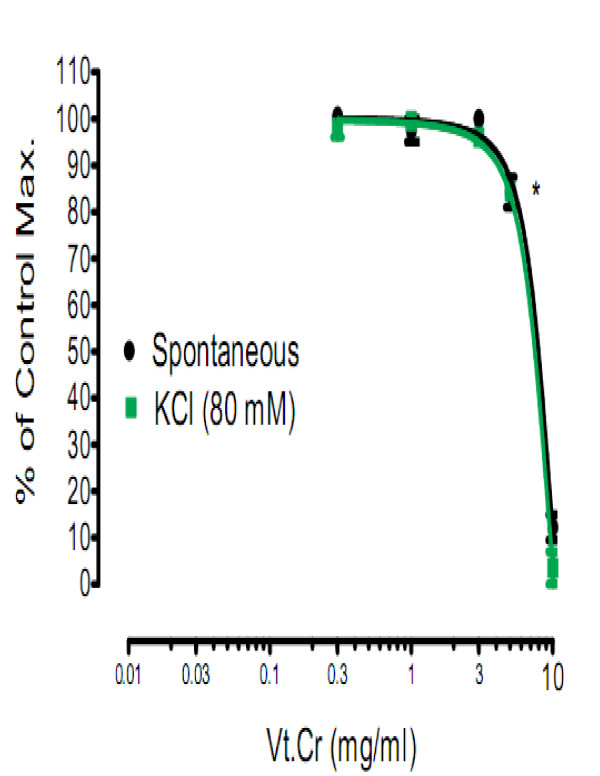
The effect of *Verbascum thapsus *extract on spontaneous and KCl-induced contractions (Values represent the mean ± SEM, n = 6, *P < 0.05 *vs*. control maximum).

**Figure 2:**
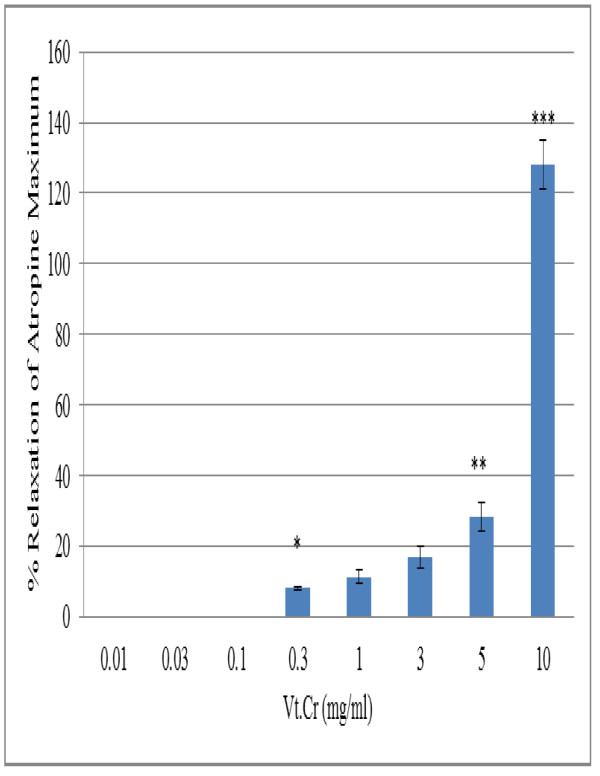
The relaxation activity of *Verbascum thapsus *on rabbit jejunal preparations expressed as a percent of the atropine maximum (Values represent the mean ± SD, n = 5,*P < 0.05, ** *P <*0.01 and ***P <0.001*vs*. atropine maximum).

Tests conducted on preparations of rabbits' jejunum showed that the Vt.Cr produced a concentration dependent relaxation of spontaneous contractions (Figure 1). The mean EC_50 _values obtained for spontaneous and KCl induced contractions were 7.5 ± 1.4 mg/ml (6.57-8.01, n = 6, *P *< 0.001 *vs*. control), and 7.9 ± 0.41 mg/ml (7.44-8.46, n = 6), respectively. The relaxation effects of the Vt.Cr were quantified against atropine; the values obtained were 11.42 ± 2, 17.0 ± 3, 28.5 ± 4, and 128.0 ± 7% (*P *< 0.001) of the atropine maximum at its corresponding concentrations, i.e., 1.0, 3.0, 5.0 and 10 mg/ml (Figure 2). Intracellular and extracellular stores of calcium exchange with each other through voltage operated calcium channels that help in regulating spontaneous intestinal responses. It is noteworthy that the contractile effects in the intestine are due to free cytosolic calcium levels that enter into sarcoplasmic reticulum through voltage operated calcium channels [26]. Thus, blocking calcium exchange through voltage operated channels relaxes intestinal tissues. Our results suggests that the plant's mode of action is possibly mediated through the calcium channels, as high molar KCl induced contractions are usually, though not necessarily, through calcium channels [25-27].

**Figure 3:**
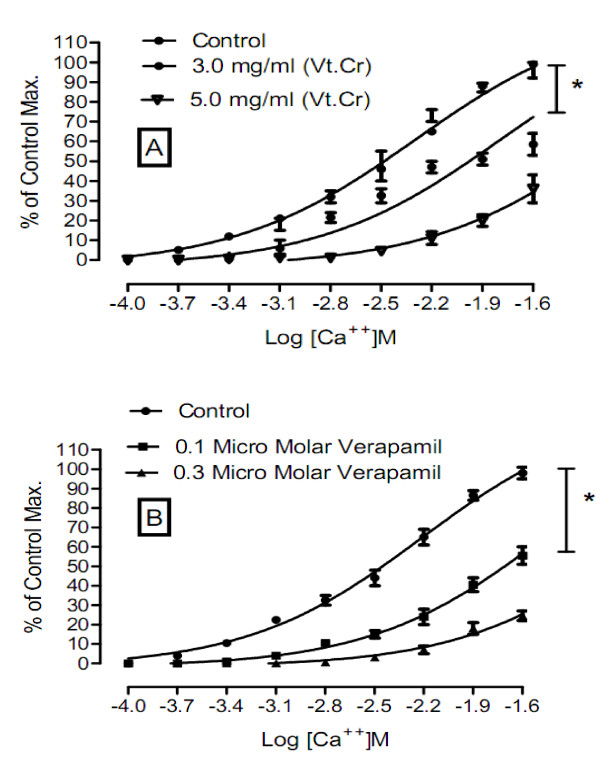
**(A) Calcium chloride curves in the presence and absence of *Verbascum thapsus *extract**. (B) Calcium chloride curves in the presence and absence of verapamil. Values represent the mean ± SEM, n = 6, *P < 0.05.

To further investigate the plant's mode of action, calcium chloride curves were constructed (Figure 3). Vt.Cr at a concentration 3 mg/ml shifted the calcium curves to right, with a mean EC_50 _value of -1.9 ± 0.06 (-1.87 - -1.98, n = 6) *vs*. the control EC_50 _= -2.5 ± 0.12 (-2.37 - -2.56, n = 6) log molar [calcium] (*P <*0.0001). Such a shift might indicate that some of the calcium channels were blocked and calcium could not enter the cell through voltage sensitive L type calcium channels [24-27]. Similarly, the calcium curve constructed for verapamil (0.1 μM) gave an EC_50 _(log molar [calcium]) value of -1.7 ± 0.1 (-1.6 - -1.8, n = 6, *P *= 0.0004) *vs*. the control EC_50 _value of -2.4 ± 0.09 (-2.3 - -2.47, n = 5). The right shift derived from the Vt.Cr samples resembled the right shift observed on the verapamil curves. We conclude, therefore, that the mode of jejunal relaxation observed might be mediated by voltage sensitive L- type calcium channels. Hence, *V. thapsus *is a potentially useful species for isolation of medicinal bioactive molecules.

## Conclusions

The present research work confirms the intestinal relaxation and anthelmintic properties of *V. thapsus*, thus supporting its use for management of abdominal pain and parasitic worms by some local tribes within the Malakand region of Pakistan.

## Abbreviation

Vt.Cr: Aqueous methanolic extract of *Verbascum thapsus*

## Competing interests

The authors declare that they have no competing interests.

## Authors' contributions

NA participated in data collection and interpretation of the relaxation activity experiments and preparation of the manuscript. SWA assisted with data collection for the anthelmintic activity experiments and writing the introduction section of the manuscript. IS and GA assisted with data collection. MG assisted with data collection and maintenance of laboratory animals. IK participated with data collection. WA assisted in copy editing of the manuscript and proof reading. All authors read and approved the final manuscript.

## Pre-publication history

The pre-publication history for this paper can be accessed here:

http://www.biomedcentral.com/1472-6882/12/29/prepub

## References

[B1] Rahmatullah Q Bhatti GR Taxonomy of scrophulariaceae from Nara desert, Pakistan Pak J Bot 2008 40 3 973 978

[B2] Faik AK Zeki A Revision of the Genus Verbascum L. (Group A) in Turkey Bot Res J 2008 1 1 9 32

[B3] Gvazava LN Kikoladze VS Verbascoside from *Verbascum phlomoides* Chem Nat Comp 2007 43 Suppl 6 710 711

[B4] Alper S Basaran D Antimicrobial activity of the leaves of *Verbascum sinuatum *L. on microorganisms isolated from urinary tract infection Afr J Microb Res 2009 3 Suppl 11 778 781

[B5] Barbara K Hydroxycinnamoyl ester glycosides and saponins from flowers of Verbascum phlomoides Phytochem 1996 34 Suppl 6 1281 1284 10.1016/s0031-9422(96)00446-3 8987909

[B6] Gvazava LN Kikoladze VS Iridoids from *Verbascum phlomoides *and *V. densiflorum* Chem Nat Comp 2009 45 Suppl 5 751 752

[B7] Basaran D Ahmet G Antimicrobial activity of some endemic *Verbascum, Salvia*, and *Stachys *Species Pharma Biol 2004 42 Suppl 4-5 301 304

[B8] Sultan MW Altaf AD Jehandar S Common medicinal plants of Chapursan Valley, Gojal II, Gilgit-Pakistan J Res Sci 2004 15 Suppl 1 41 43

[B9] Muhammad H Sumera A Mir AK Ethnopharmacology, indigenous collection and preservation techniques of some frequently used medicinal plants of Utror and Gabral, District Swat, Pakistan Afr J Trad Cam 2006 3 Suppl 2 57 73

[B10] Sheikh SA Medicinal wild plants from Lahore-Islamabad motorway (M-2) Pak J Bot 2007 39 Suppl 2 355 375

[B11] Muhammad IS Mir AK Folk use of medicinal herbs of Margalla Hills, National Park, Islamabad J Ethnopharmacol 2000 69 Suppl 1 45 56 10661883 10.1016/s0378-8741(99)00135-x

[B12] Ghulam MS Mir AK Common medicinal folk recipes of Siran Valley, Mansehra, Pakistan Ethnobot Leaf 2006 10 Suppl 1 49 62

[B13] Mahesh K Yash P Anand VK An ethnobotanical study of medicinal plants used by the locals in Kishtwar, Jammu and Kashmir, India Ethnobot Leaf 2009 13 Suppl 10 1240 56

[B14] Ilker U Suleyman B Nurettin Y Yunus D The investigation and quantitative ethnobotanical evaluation of medicinal plants used around Izmir province, Turkey J Med Pl Res 2009 3 Suppl 5 345 367

[B15] Rizwana AQ Mushtaq A Asad GM Indigenous knowledge of some important wild plants as a folk medicines in the area of Chhachh (Distt. Attock) Punjab, Pakistan EJEAFChe 2007 6 Suppl 11 2500 2511

[B16] Rosenthal PJ Katzung BG, Masters SB, Trevors AJ Tata Clinical Pharmacology of the anthelmentic drugs Clinical Pharmacology 2009 11 New Delhi: Mc Graw Hill publishers 923 924

[B17] Sofowara A Medicinal plants and Traditional medicine in Africa 1993 Spectrum Books Ltd, Ibadan, Nigeria 289

[B18] Trease GE Evans WC Ellen Green Pharmacognosy 2003 15 Saunders, an imprint of Elsevier Science Limited, printed in china 137

[B19] Harborne JB Phytochemical methods 1973 Chapman and Hall, Ltd, London 49 188

[B20] Ajaiyeoba EO Onocha PA Olarenwaju OT In vitro anthelmintic properties of *Buchholzia coriaceae *and *Gynandropsis gynandra *extract Pharm Biol 2001 39 Suppl 3 217 220

[B21] Vigar Z Atlas of Medical Parasitology 1984 2 P. G. Publishing House, Singapore 242

[B22] Dash GK Suresh P Kar DM Ganpaty S Panda SB Evaluation of Evolvulus alsinoides Linn. for anthelmintic and antimicrobial activities J Nat Rem 2002 2 Suppl 2 182 185

[B23] Shivkumar YM Kumar VL Anthelmintic activity of latex of *Calotropis procera* Pharma Biol 2003 41 Suppl 4 263 265

[B24] Gilani AH Bukhari IA Khan RA Arif-ullah K Farman U Viqar UA Cholinomimetic and calcium channel blocking activities of *Carthamus oxycantha* Phytother Res 2005 19 Suppl 8 679 683 16177970 10.1002/ptr.1727

[B25] Niaz A Shah SWA Spasmolytic Activity of Fruits of *Tamarindus indica *L JY Pharma 2010 2 Suppl 3 261 264 10.4103/0975-1483.66805 PMC2964778 21042482

[B26] Niaz A Shah SWA Antispasmodic activity of *Teucrium stocksianum *Bioss Pak J Pharm Sci 2011 24 Suppl 2 171 174 21454166

[B27] Shah SWA Samir K Waqar A Niaz A Spasmogenic, spasmolytic and antihypertensive activity of *Forsskalea tenacissima *L Afr J Pharm Pharmacol 2010 4 Suppl 6 381 385

